# Effect of residence on mothers’ health care seeking behavior for common childhood illness in Northwest Ethiopia: a community based comparative cross – sectional study

**DOI:** 10.1186/1756-0500-7-705

**Published:** 2014-10-08

**Authors:** Yalemzewod Assefa Gelaw, Gashaw Andargie Biks, Kefyalew Addis Alene

**Affiliations:** Institute of Public Health, College of Medicine and Health Science, University of Gondar, Gondar, Ethiopia

**Keywords:** Health care seeking behavior, Mothers, Urban and rural communities, Northwest Ethiopia

## Abstract

**Background:**

Children are at higher risk of acquiring infections and developing severe disease. This study assessed the health care seeking behavior and associated factors of urban and rural mothers for common childhood illness in Northwest Ethiopia.

**Methods:**

A comparative community based cross-sectional study was conducted among urban and rural mothers living in the district. A multistage sampling technique was used to select the study participants. A pre-tested and structured questioner via interview was used to collect the data. Binary logistic regression analysis was used to identify associated factors. Odds ratio with 95% CI was computed to assess the strength of the associations.

**Result:**

A total of 827 (274 urban and 553 rural) mothers were interviewed. Among these, 79.3% (95% CI: (76.5%, 82.06%)) of the mothers were sought health care in the district. Health care seeking behavior was higher among urban mothers (84.6%) than rural mothers (76.7%). Marital status, completion health extension package, and sex of child were significantly associated with health care seeking behavior of urban mothers. Whereas age of child, age and occupation of mothers, educational level of fathers, wealth quintile, and type of reported illness were significantly associated with rural mothers. Perceived severity of illness was significantly associated with both urban and rural mothers for health care seeking behavior.

**Conclusion:**

The overall health seeking behaviors of mothers for common childhood illness was high. However, urban mothers seek health care more than rural. Socio Economic position and types of reported illness has an effect for health seeking behavior of rural mothers. Whereas child sex preference and graduation status for health extension package has an effect for health care seeking behavior of urban mothers. Work on strengthen accessibility of health care services in the rural mothers and increase awareness of mothers about the disadvantage of sex preferences will improve the health care seek behavior of families regardless of the severity of illness and types of illnesses.

## Background

Millions of mothers and their children through the world are living in a social environment that doesn’t encourage health care seeking behavior [1]. Reduction of childhood mortality is a worldwide health priority and one of the Millennium Developmental Goals (MDG). According to the United Nations Children’s Fund (UNICEF) 2012 report globally 6.9 million children die before the age of five years annually. The majority of these death occurred in Sub-Saharan Africa. Ethiopia is one of the countries with a consistently high under- five mortality rate. It is ranked 36th among Sub- Saharan Africa countries [2].

Rural and urban populations have differences in socio- demographic, socio-economic and cultural compositions which have an effect on their health care seeking behavior. In sub Saharan Africa, child mortality is higher among rural, poor and less educated families [3]. Early health care seeking behavior of mothers has a potential to reduce child mortality particularly in developing countries like Ethiopia [4]. However, different studies showed that mothers health care seeking behavior for common childhood illness are influenced by socio-demographic and cultural, educational factors. Studies also indicated that timely decision to seek remedies, and times of health seeking after the onset of illness are influenced by residence [5–7]. Children from rural communities often have the highest risk of infection and severe disease especially for diarrhea, fever and acute respiratory illness (ARI), mothers are less likely to seek appropriate health care for them as compared to urban mothers [8]. Little is known about the magnitude and determinants of health care seeking behaviors of mothers’ for their children when they ill in Ethiopia especially in the study area. However, recent evidence indicates that health care seeking behaviors of mothers are increasing because of it considers only the urban dwellers.

Thus, this study aimed to identify the prevalence of health care seeking behaviors and associated factors of health care seeking behaviors among mothers for their children living in urban and rural areas.

## Methods

A community based comparative cross – sectional study was conducted in Bure district from April to June, 2103. It is about 419 km away from Northwest of Addis Ababa, the capital city of Ethiopia. According to the 2007 census projection, the district has a total population of 161,480. It has five urban and twenty three rural *kebeles* [9] *.*

Multistage sampling technique was used to select the study participants. The district was classified into two strata; urban and rural. Then three urban and six rural *kebeles* were randomly selected. For the purpose of this study, census was conducted in the selected kebele to estimate the approximate number of eligible mothers. Mothers who had under – five children with common childhood illnesses two weeks preceding the data collection period were included. Whenever there was more than one mother having under - five children in the household, one of them were selected using lottery method. The sample size was calculated using a two population proportion formula; considering 37.2% proportion of urban mothers and 22.4% rural mothers seeking health care for fever according to EDHS report [10], a 5% significance level, power of 80%,1:2 urban to rural ratio, design effect 2 and 10% possible non – response rate. The sample size was estimated to be 786 (274 urban and 553 rural). While a sample size was regionally computed to be 786, a preliminary survey prior to a data collection shows that 886 mothers were eligible.

Data were collected via interview with a pre-tested and structured questionnaire. Data collectors and supervisors were health professionals. Training was given to the data collectors and supervisors on the objective, confidentiality of information, respondent’s right and techniques of interview prior to data collection.

Socio – demographic data; sex, age, occupation, religion, educational level, and wealth quintiles were collected. Wealth quintile was collected by asking the presence or absence of durable assets and services at the house hold level. Different indicators were used for urban and rural areas to measure wealth quintiles. A score of 1 was given if the asset was present and 0 if absent. The asset mean scores were re categorized into five different wealth quintiles of equal proportion (Lowest, Second, Middle, Fourth and Highest wealth control groups) by using Principal Component Analysis (PCA).

Health care seeking behavior was the outcome variable which was dichotomized as “yes” (seeking health care) or “no” (did not seeking health care) when their children were ill. Mothers were considered as seeking health when they visited any health facilities (Hospital, Health Center, Health Post, Privet Clinic) after perceiving the illness of their child within two weeks of data collection period.

The returned questionnaires were checked for completeness by the investigator. The data were entered into Epi INFO version 3.5.3 and analyzed by SPSS version 20. Descriptive statistics were done to summarize the data in relation to the different variables. Binary logistic regression model was used to assess the association between the dependent and independent variables. Variables having P value ≤0.2 in the bivariate analysis were entered into multivariate logistic regression models to control the effect of confounding. Both Crude Odds Ratio (COR) and Adjusted Odds Ratio (AOR) with 95% confidence interval (CI) were calculated to measure associations.

Ethical clearance was obtained from the institutional review board of the University of Gondar. A formal letter of communication to the district health office was secured. Verbal informed consent was obtained from each study participants prior to the interview. They were also informed that participation was on voluntary basis and they can withdraw at any time if they are not comfortable about the interview.

## Results

### Socio-demographic characteristics of participants

A total of 827 mothers (with a response rate of 93.3%) were interviewed. Nearly one third (33.1%) were urban dwellers. Among the study participants only 94(34.3%) urban and 73(13.2%) rural mothers were attended formal education. The mean age (SD) of the mothers children was 31.4 (6.2) years and 23.6 (15.7) months, respectively. Seventy - seven (28.1%) of urban and 430 (78%) of rural households had graduated from the health extension package in the community.

### Health care seeking behaviors

The overall health care seeking behavior of mothers for common childhood illness was 79.3% (95% CI: (76.5%, 82.06%)). A total of 84.6%: (95% CI: (80.3%, 88.8%)) urban mothers and (76.7%: (95% CI: (73.2%, 80.2%)) rural mothers were seeking health care for common childhood illness.

Governmental health center and hospitals were the most common place where mothers sought health care [79.1%: (80% urban and 54.3% rural)]. The main reasons reported for not seeking health care were the thought that the disease may resolve by itself over time [Table 1].

**Table 1:** **Health care-seeking behaviors of mothers for common childhood illness at urban and rural residence, Bure district, Northwest Ethiopia, 2013**

Variable	Urban	Rural	Total
	Number (%)	Number (%)	Number (%)
**Reported illness**			
Diarrhea	113(41.3%	234(42.3%)	347(42%)
Fever	103(37.6%)	150(27.1%)	253(30.6%)
ARI	58(21.2%)	169(30.6%)	227(27.4%)
**Health care seeking behavior**			
Home	1(0.4%)	30(5.4%)	31(3.7%)
Traditional healers	2(0.7%)	17(3.1%)	19(2.3%)
HC/Hospital	219(80%)	300(54.3%)	519(62.8%)
Health post	1(0.4%)	104(18.8%)	105(12.7%)
Private	12(4.4%)	20(3.6%)	32(3.9%)
Not seek at all	39(14%)	82(14.8%)	121(14.6%)
**Health care seeking behavior at HF**			
No	42(15.3%)	129(23.3%)	171(20.7%)
Yes	232(84.6%)	424(76.7%)	656(79.3%)
**Time of health seeking after onset of the illness**			
First day	96(41.4%)	80(19%)	176(27%)
Between 1st and 2nd day	85(36.6%)	103(24.3%)	188(28.6%)
Between 3rd and 4th day	43(18.5%)	181(42.7%)	224(34%)
After 4th	8(3.5%)	60(14%)	68(10.4%)
**Reasons for not seeking health care**			
Perceived resolves by itself	37(34%)	64(66%)	97(56.7%)
Lack of money	13(31.7%)	28(68.3%)	41(24%)
Illness was not sever enough	1(3.2%)	30(96.8%)	31(18%)
Did not trust by treatment	2(11.1%)	16(88.9%)	18(10.5%)
Distance of HF	2(22.2%)	7(77.8%)	9(5.3%)

### Factors associated with health care seeking behaviors

We have fitted three different models to assess health care seeking behavior. The first model was fitted to assess the overall factors of health care seeking behavior for common child hood illness. Variables such as residence, perceived severity, marital status, health extension package graduation status, sex and age of child’s, age and occupation of mothers, educational level of fathers, wealth quintiles, and types of reported illness were significantly associated with health care seeking behaviors for the whole mothers irrespective of residence. Mothers who lived in urban [AOR: 11.5, 95% CI (5.407, 24.483)], and perceived severity of illness [AOR: 9.23, 95% CI (5.88, 14.562] were more likely to seek health care than the counter parts. Mothers who graduated from health extension package 96.6% times [AOR: 0.034, 95% CI (0.016, 0.074)] and male sex preference children 61.0% [AOR: 1.610, 95% CI, (1.040, 2. 495)] were more likely to seek health care when compared to the counterpart. Mothers in the fourth wealth Quintiles were 5.126 times more likely to seek health care than those in the lowest Quintiles [AOR: 5.126 95% CI: (2.544, 10.331)].

Furthermore, mothers’ whose husband attend formal education were 2.467 times more likely to seek health care than those mothers whose husband didn’t attend formal education (AOR: 2.467, 95% CI: (1.385, 4.380)) [Table 2].

**Table 2:** **Factors associated with health care seeking behaviors of mothers’ for childhood illness, Bure district, Northwest Ethiopia, 2013**

Variables	Health care seeking behavior		COR (95% CI)	AOR (95% CI)
	Yes	No		
**Age of mothers’ in year**				
≤ 30 years	380	76	1	
≥ 31 years	276	95	0.58(0.414,0.816)	
**Occupation of mothers’**				
Farmer	318	112	1	
Merchant	107	14	2.692(1.481,4.891)	
House wife	231	45	1.808(1.230,2.658)	
**Occupation of husband (672)**				
Farmer	358	100	1	
Merchant	108	21	1.437(0.856,2.410)	
Governmental	35	7	1.397(0.602,3.239)	
Others**^**	39	4	2.723(0.951,7.803)	
**Residence**				
Rural	424	129	1	1
Urban	232	42	1.681(1.146,2.465)	11.501(5.407, 24.483)^**^
**Perceived severity**				
Not perceived sever	189	125	1	1
Perceived sever	467	46	6.714(4.601 ,9.798)	9.258(5.88 , 14.562)^**^
**Health extension package**				
Graduated	572	96	1	1
un graduated	84	75	0.188(0.129,0.275)	0.034(0.016 , 0.074)^**^
**Type of reported illness**				
Diarrhea	288	59	1	1
Fever	207	46	0.922(0.603,1.410)	1.197(0.695, 2.031)^1^
ARI	161	66	0.500(0.335,0.746)	0.623(0.375, 1.037)^1^
**Age of children in month**				
0 – 11 month	180	58	1	
12 – 23 month	144	23	2.017(1.187,3.429)	
24 – 35 month	151	40	1.216(0.770,1.922)	
36 – 47 month	78	29	0.867(0.516,1.456)	
48 – 59 month	103	21	1.580(0.907,2.757)	
**Sex of the child**				
Female	340	102	1	1
Male	316	69	1.374(0.976,1.934)	1.610(1.040 , 2. 495)^*^
**Knowledge status of mothers’**				
Poor	355	104	1	
Good	301	67	1.316(0.934,1.855)	
**Educational status of husband**				
No formal education	358	104	1	1
Formal education	182	28	1.888(1.199,2.973)	2.467(1.385 , 4.380)^*^
**Wealth quintiles**				
Lowest	119	51	1	1
Second	132	31	1.825(1.095,3.041)	1.561(0.845 , 2.885)
Middle	144	23	2.683(1.550,4.646)	5.126(2.544 , 10.331)^**^
Fourth	136	36	1.619(0.989,2.469)	1.720(0.897 , 3.297)
Highest	125	30	1.786(1.066,2.992)	1.644(0.843 , 3.205)

Note: **p-value ≤0.001, *p-value ≤0.005, ^1^Marginally significance at p = 0.05; **^** daily laborer.

The second model was fitted only for rural mothers. Accordingly, age and occupation of mothers’, educational level of fathers, wealth Quintiles, age of child’s, types of reported illness showed a significant association. Rural mothers with relatively younger age were *1.79* times more likely to seek health care as compared to older age mothers [AOR: 0.558, 95% CI, (0.339, 0.919)] and mothers who had younger children were 4 times more likely to seek health care as compared to mothers who have older children [AOR: 0.240, 95% CI: (0.083, 0.699)]. In addition, mothers who are in third and fourth wealth quintiles were also more likely to seek health care compared to in the lowest Quintiles [AOR: 4.456, 95% CI: (2.091, 9.965)] and [AOR: 2.522, 95% CI: (1.186, 5.365)], respectively.

Mothers who had formal education attended husbands [AOR: 3.170, 95% CI: ( 1.490, 6.746)] and a child with diarrheal disease [AOR: 0.538, 95% CI: ( 0.306, 0.946)] were more likely to seek health care as compared to their counterparts. Moreover, mothers’ who gave birth in the health center [AOR: 0.484, 95% CI: (0.257, 0.913)] and hospital [AOR: 0.328, 95% CI: (0.128, 0.890)] seek health care more likely than those who give birth at home [Table 3].

**Table 3:** **Factors associated with health care seeking behaviors for rural mothers, Bure district, Northwest Ethiopia, 2013**

Variables	Health care seeking behavior		COR (95% CI)	AOR (95% CI)
	Yes	No		
**Age of mothers**				
≤ 30 years	216	46	1	1
≥ 31 years	208	83	0.534(0.355,0.802)	0.558(0.339,0.919)^*^
**Perceived severity**				
perceived mild	96	88	1	1
perceived sever	328	41	7.33(4.747,11.328)	12.477(7.269,21.415)**
**Type of reported illness**				
Diarrhea	187	47	1	1
Fever	120	30	1.005(0.602,1.678)	1.648(0.874,3.109)
ARI	117	52	0.566(0.358,0.893)	0.538(0.306,0.946)^*^
**Education of mothers**				
No formal education	363	117	1	
Formal education	61	12	1.638(0.853,3.149)	
**Occupation of mothers**				
Farmer	306	111	1	
Merchant	59	9	2.378(1.141,4.955)	
Others^	56	9	2.378(1.141,4.955)	
**Place of child’s birth**				
Home	303	82	1	1
Health center	85	27	0.852(0.518,1.400)	0.484(0.257,0.913)*
Health post	16	7	0.619(0.246,1.554)	0.451(0.128,1.583)
Hospital	20	13	0.416(0.199,0.872)	0.328(0.128,0.890)*
**Age of child in month**				
≤ 11	122	49	1	
12 – 23	76	11	2.775(1.359,5.666)	1.400(0.459,4.26)
24 – 35	102	29	1.413(0.832,2.398)	0.413(0.154,1.102)
36 – 47	44	22	0.803(0.437,1.478)	0.240(0.083,0.699)^**^
48 – 59	80	18	1.785(0.971,3.283)	0.641(0.220,1.871)
**Immunization status of child**				
Completed	311	81	1	
Started	72	36	0.521(0.326,0.832)	0.254(0.095,0.684)*
Defaulted and Unimmunized	41	12	0.890(0.447,1.771)	0.674(0.240,1.897)
**Education of husband (352)**				
No formal education	257	91	1	1
Formal education	95	11	3.058(1.567,5.967)	3.170(1.490,6.746)*
**Wealth Quintiles**				
Lowest	75	39	1	1
Second	80	26	1.64(0.912,2.949)	1.332(0.651,2.725)
Middle	82	17	2.906(1.524,5.539)	4.456(2.091,9.965^)**^
Fourth	95	26	1.840(1.028,3.249)	2.522(1.186,5.365)^*^
Highest	92	21	1.981(1.069,3.671)	2.092(0.967,4.526)

Note: **p-value ≤0.001, *p-value ≤0.005; significance at 5% level of significance.

^shows the job distributions of others since it is greater than 5%.

The third model was fitted for urban mothers only. As a result, urban mothers who were married were *1.7* times more likely to seek health care than unmarried mothers’ [AOR: 0.228, 95% CI: (0.027, 0.719)]. Mothers who had male children seek health care 6.2 times more likely than those who had female children [AOR: 6.222, 95% CI: (2.263, 17.109)]. Mothers who were graduated for health extension package were *97.6%* times more likely to seek health care than the counterpart [AOR: 0.024, 95% CI: (0.007, 0.085)] [Table 4].

**Table 4:** **Factors associated with health care seeking behaviors of urban mothers, Bure district, Northwest Ethiopia, 2013**

Variables	Health care seeking behavior		COR (95% CI)	AOR (95% CI)
	Yes	No		
**Marital status**				
Married	188	30	1	1
Not married	44	12	0.585(0.278,1.233)	0.228(0.072 , 0.719)^*^
**Perceived severity**				
perceived mild	93	37	1	1
perceived sever	139	5	11.060(4.192,29.179)	32.925(9.469,114.486)^**^
**Health extension package**				
Graduated	33	9	1	1
Not graduated	84	148	0.155(0.71,0.339)	0.024(0.007,0.085)^**^
**Reported types of illness**				
Diarrhea	101	12	1	
Fever	87	16	0.646(0.290,1.440)	
ARI	44	14	0.373(0.160,0.872)	
**Sex of the child**				
Female	11	31	1	1
Male	120	112	3.019(1.449,6.294)	6.222(2.263,17.109)^**^
**Husband occupation (188)**				
Farmer	95	10	1	
Merchant	49	10	0.516(0.201,1.323)	
Governmental	44	10	0.463(0.180,1.194)	

Note: **p-value ≤0.001, *p-value ≤0.005; significance at 5% level of significance.

## Discussion

Seeking health care for common childhood illnesses from health institution has a great potential to reduce child mortality [4]. In this study, health care seeking behavior of mothers was 79.3% (95% CI: 76.5%, 82.06%). There was a significant difference of health care seeking behaviors between rural (76.7%) and urban mothers (84.6%). Urban mothers were 11.5 times more likely to seek health care than rural mothers. This might be due to the accessibility of health care for urban mothers. In Ethiopia, health care coverage is relatively high in urban than rural [11]. It could be also due to accessibility of information about the importance of seeking of health care for childhood illness.

Both urban and rural mothers sought health care for diarrheal illness than ARI. This finding was similar with other study done in Bahir Dar and EDHS 2011 report [10, 12]. This could be due to the reason that diarrhea is more visible for the mothers than ARI. And it could be difficult for uneducated mother to feel the severity of fever and ARI. But it is inconsistent with a study done in Derra district, Ethiopia [5] and Rural Uganda [13] in which they sought health care more for fever.

This result showed that residence has a great effect in the mothers’ health care seeking behavior for common childhood illness. The study result is consistent with similar studies done in Ethiopia [5, 11] and, in Africa [14, 15]; South Africa (14) and South East Nigeria [15]. The time of seeking health care after the perception of illness was also significantly differ between urban and rural mothers. Urban mothers’ seek health care on the first day of perceived onset of illness than rural mothers’. The possible reason for delaying of rural mothers to seek health care might be their attempt to try home remedies, lack of money, or socio – cultural factors like consulting the elders and relatives.

The main factors influencing mothers’ health care seeking behavior of the district identified in this study were perception of severity of illness, graduation of health extension package, type of reported illnesses, sex of the child, educational status of husband and wealth quintile.

Even though, the principle of health care service delivery system of Ethiopia assumed that health care seeking behavior of mothers is equal. This study revealed that mothers’ health care seeking behaviors were more likely influenced by perception of severity illness. This is consistent with report of other studies [5, 15–20].

Poverty is a serious constraint on mothers’ choices about seeking health care. The finding of this study also indicated that socio–economic and graduation status of the household health extension package was significant predictors of health care seeking behaviors of mothers’. Studies done in Ethiopia also showed that urban inhabitants are in the highest quintiles than rural [5, 11]. But in this study, both urban and rural dwellers found in equal proportion from the lowest to the highest quintiles.

Mothers who had male children were more likely to seek health care than who had female. This is supported by a study done in Ethiopia and Kenya [11, 18]. Though, other studies done in rural parts of Nigeria and India reported that sex had no association with mothers’ health care seeking behavior [18, 19]. This is due to the fact that in Ethiopia there is a cultural influence in which mother are giving priority for male child than female child. Moreover, the starting of health extension program was lately in urban areas can be a reason for the difference [10].

The present studies also documented that husband’s educational status had a significant effect on mothers’ health care seeking behavior.

This study has the following limitation: first the study didn’t include other child hood illness though they are not common in the study area. Second, as it needs longer time to see the seasonal variation they didn’t consider the seasonal variation as independent variables.

## Conclusion

Residence has an effect on the mothers’ health care seeking behaviors for common childhood illness. Urban mothers seek health care more than rural mothers. Age and occupation of the mothers’, educational level of the fathers, wealth quintiles, age of child, types of reported illness showed a significant association for health seeking behavior of rural mothers for common child hood illness. Whereas marital status, graduation status for health extension package and sex of children’s showed a significant association for health care seeking behavior of urban mothers. It is better to give infancies especially for rural mothers to seek health care regardless of the severity of illness and types of illnesses.
